# Prospective study of determinants and costs of home births in Mumbai slums

**DOI:** 10.1186/1471-2393-10-38

**Published:** 2010-07-30

**Authors:** Sushmita Das, Ujwala Bapat, Neena Shah More, Latika Chordhekar, Wasundhara Joshi, David Osrin

**Affiliations:** 1 Society for Nutrition, Education and Health Action (SNEHA), Urban Health Centre, Chota Sion Hospital, 60 Feet Road, Shahunagar, Dharavi, Mumbai 400017, Maharashtra, India; 2 UCL Centre for International Health and Development, Institute of Child Health, 30 Guilford St, London WC1N 1EH, UK

## Abstract

**Background:**

Around 86% of births in Mumbai, India, occur in healthcare institutions, but this aggregate figure hides substantial variation and little is known about urban home births. We aimed to explore factors influencing the choice of home delivery, care practices and costs, and to identify characteristics of women, households and the environment which might increase the likelihood of home birth.

**Methods:**

As part of the City Initiative for Newborn Health, we used a key informant surveillance system to identify births prospectively in 48 slum communities in six wards of Mumbai, covering a population of 280 000. Births and outcomes were documented prospectively by local women and mothers were interviewed in detail at six weeks after delivery. We examined the prevalence of home births and their associations with potential determinants using regression models.

**Results:**

We described 1708 (16%) home deliveries among 10 754 births over two years, 2005-2007. The proportion varied from 6% to 24%, depending on area. The most commonly cited reasons for home birth were custom and lack of time to reach a healthcare facility during labour. Seventy percent of home deliveries were assisted by a traditional birth attendant (*dai*), and 6% by skilled health personnel. The median cost of a home delivery was US$ 21, of institutional delivery in the public sector US$ 32, and in the private sector US$ 118. In an adjusted multivariable regression model, the odds of home delivery increased with illiteracy, parity, socioeconomic poverty, poorer housing, lack of water supply, population transience, and hazardous location.

**Conclusions:**

We estimate 32 000 annual home births to residents of Mumbai's slums. These are unevenly distributed and cluster with other markers of vulnerability. Since cost does not appear to be a dominant disincentive to institutional delivery, efforts are needed to improve the client experience at public sector institutions. It might also be productive to concentrate on intensive outreach in vulnerable areas by community-based health workers, who could play a greater part in helping women plan their deliveries and making sure that they get help in time.

## Background

If we are to improve maternal and newborn health and survival, it is generally agreed that women should be assisted during delivery by trained healthcare professionals with appropriate equipment, medications and access to referral systems [1]. The presence of such skilled birth attendants is a target under the fifth Millennium Development Goal. Although giving birth at home does not preclude domiciliary skilled care, for most of the world skilled birth attendance implies institutional delivery [2].

Cities illustrate the fact that the availability of health care does not necessarily lead to its use. Although India's National Population Policy (2000) set a goal of 80% institutional delivery by 2010, [3] more than one-third of births in urban India take place at home, with compromised hygiene and without skilled birth attendants. In slums, the proportion is closer to half, despite the proximity and multiplicity of health care providers [4]. In a recent initiative to increase skilled attendance at delivery, the Government of India has introduced a conditional cash transfer scheme, the *Janani Suraksha Yojana*, under which women who choose institutional delivery may claim a cash entitlement.

Around a thousand million people live in slums, among them almost half of the urban population of low-income countries [4]. Lack of access to adequate housing, safe drinking water, sanitation and basic health infrastructure affect the health of slum dwellers, particularly women and children. Mumbai is one of India's largest metropolises, and arguably its most dynamic. It is distinctive for its wide network of both public and private sector health providers, yet many women choose to deliver their babies at home rather than in hospitals or maternity homes. The current estimate is 14%, increasing to 17% in slum dwellers [5]. Little is known about the levels and determinants of home delivery in a megacity where health care resources are plentiful and their uptake seemingly ubiquitous, although the literature on maternity care for the urban poor is growing [6-11]. This paper draws information from a community-based maternity surveillance system, covering a population of about 280 000 in slum areas. Maternity experience was documented for all women living in the sample areas, as part of the City Initiative for Maternal and Newborn Health [12]. We have described inequalities in maternal and newborn health in this population, [13] and the pathways followed in routine maternity care [14]. We wanted to explore factors influencing the choice of home delivery, care practices and costs. We also wanted to identify characteristics of women, households and the environment which might increase the likelihood of home birth.

## Methods

### Study location and population

The capital of Maharashtra state, Mumbai is India's most populous city. According to the Census of 2001, slums are home to 54% of the city's 16.4 million people [15]. The Municipal Corporation of Greater Mumbai provides public sector health services across 24 urban wards in three zones: city, central and western. The Corporation's Department of Public Health administers three tertiary medical colleges, five specialist hospitals, 12 peripheral general hospitals, 24 maternity homes, 168 dispensaries, and 167 health posts.

The study involved a descriptive analysis of determinants of home births over two years in 48 vulnerable urban clusters. It also examined the expenditure involved in delivery care. The surveillance system from which data were drawn has been described elsewhere [12,13]. Briefly, a prospective, key informant vital registration system was set up to identify births, stillbirths, neonatal deaths, infant deaths, and maternal deaths. The sampling frame included the most vulnerable areas of slums in six municipal wards (F North, G North, H East, K West, M East and P North). These were selected purposively for accessibility and a range of infant mortality rates according to Municipal Corporation estimates. Each cluster consisted of 1000-1500 households; some clusters included entire slum areas, while others were subdivisions of larger geographical areas. Eighteen out of the 48 areas involved in the study were situated on or beside hazardous locations like railway lines, garbage dumps and polluted bodies of water. A substantial proportion of households did not have metered electric supply (28%), access to individual or communal piped water (21%) or individual toilet facilities (94%). Twenty-six percent of houses were of insubstantial construction (data from City Initiative, unpublished).

### Procedures

Vital events were identified by 99 locally resident women, generally two per cluster, each covering an average 600 households. Events were confirmed by 12 interviewers, each responsible for four clusters, who visited women and arranged an interview at about six weeks after delivery. After an explanation of the purpose of the study, participants were asked for verbal consent to interview and assured of the confidentiality of data. The interview was based on a predominantly closed questionnaire with modules on demography and socioeconomic factors, maternal history, antenatal, delivery, postnatal, and newborn care, illness and care-seeking. In the event of a home birth, we sought information on reasons for delivering at home, the primary birth attendant, and hygiene during and immediately after birth. We also asked about complications during childbirth and subsequent care-seeking patterns. In an adjunct sub-study, we collected data on expenditure with an additional questionnaire for all respondents over a three-month period. We asked about direct and indirect expenditure - itemised and total - in the antenatal, intrapartum and postnatal periods, either at home or at a health facility.

### Statistical analysis

We used asset scores, ordered and divided by quintiles, to describe socioeconomic status. Individual scores were assigned to respondents on the basis of standardised weights for the first component of a principal components analysis in Stata 10 (College Station, TX, USA) [16,17]. Assets included a range of consumer durables and house ownership, house construction, possession of a ration card, source of electricity, and type of toilet. The proportion of home deliveries, reasons for them, birth attendants, and hygiene practices were summarised with frequencies and percentages, using SPSS 15.0 (SPSS Inc, Chicago, Illinois, USA). The distribution of expenditure on delivery care was positively skewed and is summarised with medians and interquartile ranges. Determinants of home delivery were examined through two-way scatter plots, tables of frequencies and proportions, and regression analysis. We used random effects logistic regression in Stata 10 to adjust for the clustered nature of the sample. Quadrature checks confirmed the acceptability of this approach.

### Ethical approval

Data for the study originated from a trial approved by the Municipal Corporation of Greater Mumbai, the Independent Ethics Committee for Research on Human Subjects (Mumbai committee, reference IEC/06/31), and the ethics committee of the Institute of Child Health and Great Ormond Street Hospital for Children.

## Results

**Figure 1:**
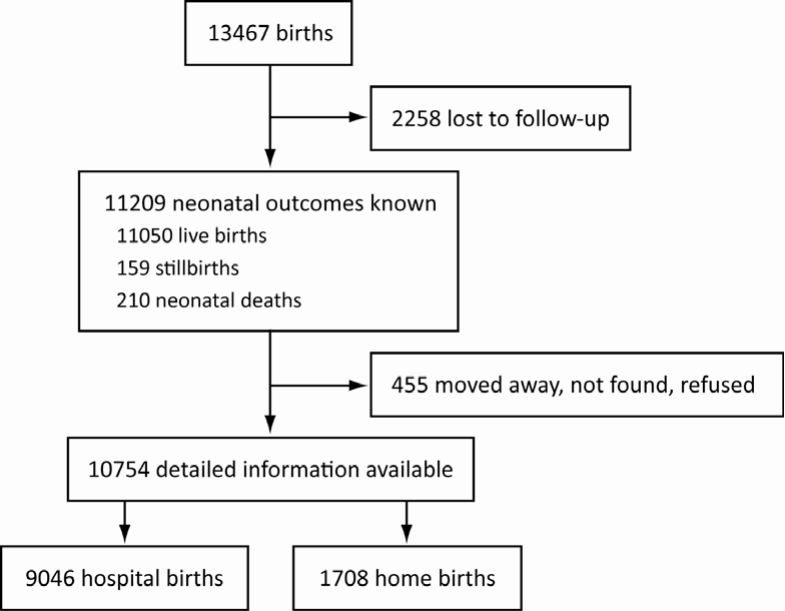
**Study flow chart**.

We identified 13 467 births from 1^st ^October 2005 to 30^th ^September 2007 (Figure 1). Neonatal outcomes were known for 11 209 births, and 2258 (17%) were lost to follow-up. The main reasons for loss were that families moved out of the study area or that women who had come to the city for maternity went back to their marital homes elsewhere. We were able to collect detailed information on 10 754 births (79%), which included 9046 institutional deliveries (77% of those identified) and 1708 home deliveries (97%).

Table 1 summarizes place of birth, for deliveries within and outside Mumbai, and by six urban wards. The ward-based figures include both women who delivered in Mumbai and women who chose to deliver elsewhere. Although the overall proportion of home deliveries was 16%, there was a substantial difference in the proportions within (10%) and outside (38%) the city. Women who left the city for delivery tended to have been living there for a shorter time (37% for less than one year; 306/829), compared with women who delivered in Mumbai (13%; 113/879). Women having their first baby were also more likely to return to places outside the city (26%; 211/829) compared with multiparous women (11%; 95/879).

**Table 1:** Frequency and proportion of institutional and home delivery in 48 Mumbai slums

**Deliveries**		**Home delivery**	**(%)**	**Institutional delivery**	**(%)**	**Total**	**(%)**
	
All		1708	(16)	9046	(84)	10754	(100)
Outside Mumbai		829	(38)	1360	(62)	2189	(100)
In Mumbai		879	(10)	7686	(90)	8565	(100)
By urban ward in which the woman resided	Mean asset rank*						
M East	(1)	530	(24)	1649	(76)	2179	(100)
F North	(3)	423	(24)	1365	(76)	1788	(100)
P North	(6)	191	(14)	1212	(86)	1403	(100)
K West	(5)	221	(13)	1463	(87)	1684	(100)
G North	(2)	231	(13)	1588	(87)	1819	(100)
H East	(4)	112	(6)	1769	(94)	1881	(100)

Data presented for 10 754 deliveries for the period 2005-2007

*Mean asset score for households interviewed in each ward, ranked from lowest (1) to highest (6)

The proportion of home deliveries also varied by city ward, from 6% (H East) to 24% (F North and M East). There was considerable heterogeneity within wards. Ranking by mean socioeconomic score for each urban ward did not suggest a relationship between home delivery and poverty, probably because a ward is too large a unit for meaningful comparison. We did not find significant differences in stillbirth or neonatal mortality rates between home and institutional deliveries (data not presented here).

Table 2 compares the profiles of 1708 women who gave birth at home and 9046 women who had institutional deliveries. The impression is that women who gave birth at home were older, less likely to have gone to school, had been married younger and had their first baby in their teens. They were also more likely to be Muslim and came disproportionately from lower socioeconomic strata. Note that in our sample the least poor women were not wealthy, representing simply the higher end of slum residents. One-third of women who had an institutional delivery were primiparous, compared with less than one-fifth who had home births.

**Table 2:** Characteristics of respondents, for home and institutional births in 48 Mumbai slums

	Home delivery		Institutional delivery		All	
	
	Frequency	(%)	Frequency	(%)	Frequency	(%)
	
Woman's age						
Under 20	153	(9)	882	(10)	1035	(10)
20-29	1264	(74)	7035	(78)	8299	(77)
30 or more	282	(17)	1129	(12)	1411	(13)
Unknown	9	(<1)	0	(0)		
Woman's age at marriage						
Under 20	1419	(83)	5988	(66)	7407	(69)
20-29	278	(16)	2990	(33)	3268	(30)
30 or more	3	(<1)	68	(1)	71	(1)
Unknown	8	(<1)	0	(0)	8	(<1)
Woman's age at first pregnancy						
Under 20	1064	(62)	4266	(47)	5330	(50)
20-29	632	(37)	4670	(52)	5302	(49)
30 or more	6	(<1)	110	(1)	116	(1)
Unknown	6	(<1)	0	(0)	6	(<1)
Parity						
First baby	306	(18)	3180	(35)	3486	(32)
Second or third baby	826	(48)	4195	(46)	5021	(47)
Fourth, fifth or sixth baby	471	(28)	1490	(17)	1961	(18)
Seventh or more	105	(6)	181	(2)	286	(3)
Woman's education						
No schooling	912	(53)	2105	(23)	3017	(28)
Primary, class 1-4	133	(8)	533	(6)	666	(6)
Secondary, class 5-7	332	(19)	2317	(26)	2649	(25)
Secondary, class 8-10	290	(17)	3230	(36)	3520	(33)
Higher secondary, class 11-12	4	(<1)	34	(<1)	38	(<1)
College or other higher education	37	(2)	827	(9)	864	(8)
Religion						
Muslim	997	(58)	3949	(44)	4946	(46)
Hindu	663	(39)	4407	(49)	5070	(47)
Other	48	(3)	690	(7)	738	(7)
Asset score quintile						
1 Lowest	616	(36)	1505	(17)	2121	(20)
2	471	(28)	1711	(19)	2182	(20)
3	310	(18)	1835	(20)	2145	(20)
4	199	(12)	1960	(22)	2159	(20)
5 Highest	112	(6)	2035	(22)	2147	(20)
Total	1708	(100)	9046	(100)	10754	(100)

Data presented for 10 754 deliveries for the period 2005-2007

**Figure 2:**
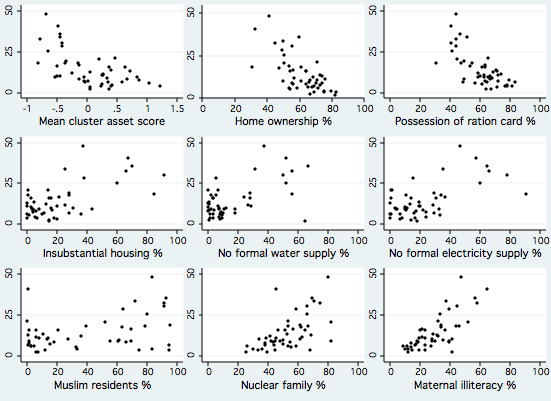
**Scatterplots of home births (%, as *y *axis) against nine independent cluster-level variables, for 10 754 deliveries in 48 Mumbai slum areas, 2005-2007**.

Figure 2 plots the proportion of home deliveries in each cluster against nine independent variables in three groups: economic (mean asset score per cluster, proportion of residents who owned their homes, and proportion of resident families who had a ration card), environmental (proportion of housing of temporary or insubstantial construction, lack of common or private water supply such that residents had to buy water from tankers, and lack of metered electricity supply necessitating access through informal channels), and demographic (proportion of residents who identified themselves as Muslim, proportion of families considered nuclear, and levels of maternal illiteracy). There is a variable, but strong, visual impression of association.

Table 3 summarises random effects logistic regression models with home delivery as a dependent variable. In moving from univariable to multivariable models, we removed five independent variables: educational level because it was related to literacy, duration of residence because it had a minimal effect on the odds, and infant sex, Muslim religion and location of the slum by a railway line as they were not associated with the outcome in univariable analysis. In the adjusted model, the odds of home delivery increased with illiteracy, parity, socioeconomic poverty, poorer housing, informal water supply, population transience, and hazardous location. Older women were more likely to have home deliveries. Antenatal care was so common (93% made at least one visit) that we chose to use the WHO recommendation of a minimum of three visits as an independent variable. Making less than three antenatal care visits was strongly associated with subsequent home delivery in univariable analysis, although the effect was attenuated by adjustment for potential confounders (with a reduction in odds ratio from 11.9 to 2.7).

**Table 3:** Univariable and multivariable random effects logistic regression models

	Univariable analysis n = 10 754			Multivariable analysis n = 10 721		
	OR	95% CI	p	OR	95% CI	p
	
*Demographic*						
Unable to read	2.66	2.37-2.98	< 0.001	1.55	1.34-1.78	< 0.001
Educational level	0.98	0.98-0.99	< 0.001			
Nuclear family	1.54	1.37-1.73	< 0.001	1.01	0.86-1.18	0.907
Maternal age	1.03	1.02-1.04	< 0.001	0.97	0.95-0.99	0.001
Parity	1.55	1.44-1.66	< 0.001	1.56	1.40-1.74	< 0.001
Infant sex	0.99	0.89-1.11	0.887			
Duration of residence	1.00	1.00-1.00	< 0.001			
Muslim	1.14	0.98-1.32	0.08			
*Socioeconomic*						
Home ownership	0.55	0.50-0.62	< 0.001	0.93	0.77-1.12	0.457
Ration card	0.50	0.44-0.56	< 0.001	0.93	0.78-1.12	0.466
Socioeconomic quintile	0.66	0.63-0.70	< 0.001	0.84	0.79-0.89	< 0.001
*Environmental*						
Temporary house (*kaccha*)	1.74	1.52-1.99	< 0.001	1.27	1.07-1.50	0.005
Informal water supply	1.38	1.19-1.60	< 0.001	1.20	0.96-1.37	0.043
Informal electricity supply	1.76	1.54-2.00	< 0.001	1.15	0.96-1.37	0.124
Annual migration > 25%	2.55	1.51-4.31	< 0.001	1.61	1.09-2.36	0.016
Residence near dump, marsh, creek	2.59	1.25-5.38	0.011	1.71	1.03-2.85	0.039
Residence by railway line	0.58	0.24-1.40	0.222			
Healthcare						
Registration for delivery	0.03	0.02-0.03	< 0.001	0.057	0.05-0.07	< 0.001
< 3 antenatal visits	11.93	10.40-13.68	< 0.001	2.73	2.27-3.27	< 0.001

Random effects logistic regression models with home delivery as the dependent variable, for

10 754 deliveries in 48 Mumbai slums, 2005-2007.

OR: odds ratio; CI: confidence interval.

Table 4 shows that the commonest reason given for home birth was custom and tradition (28%). Other common explanations included lack of time to reach a facility due to rapid progress of labour (13%), difficulty in finding someone to accompany the woman in labour to hospital (8%), and fear of hospital staff (7%). 56% of women had planned to deliver in an institution, but did not manage to get there because of rapid progress of labour (23%) or lack of a companion (12%). Perhaps surprisingly, 128 women (13%) had registered for institutional delivery, but said that they had delivered at home because it was customary. Lack of family support was a more common reason given by Muslim (17%; 154/900) than by Hindu women (10%; 69/678). This was also true for multiparous (18%; 151/884), than for primiparous women (10%; 72/734).

**Table 4:** Reasons given for home delivery in 48 Mumbai slums

Reason	Frequency	(%)
Custom	480	(28)
Labour too quick to reach institution	230	(13)
Nobody to accompany woman to institution	136	(8)
Fear of institution staff	117	(7)
Convenience	104	(6)
Hospital far from home	101	(6)
Family constraints (permission, nobody to look after children)	93	(5)
Not registered for institutional delivery	57	(3)
Financial barriers	49	(3)
Lack of transport	48	(3)
Asked to return to institution later, but delivery ensued	38	(2)
Poor perception of institutional care	25	(1)
Not admitted to institution because of insufficient documents	8	(<1)
Other	92	(5)
Missing data	130	(8)

Total	1708	(100)

The principal birth attendant at 1194 (70%) home deliveries was an informal provider (the *Dai*, or traditional attendant). Only 110 (6%) home deliveries were attended by skilled personnel such as a doctor or nurse. Most birth attendants either washed their hands (67%) or wore new gloves (11%). A new or boiled blade was used to cut the umbilical cord after 1593 (93%) deliveries, and the thread used to tie the cord had been boiled in half of cases. Boric powder (39%) and turmeric (18%) were the most popular cord dressings.

Table 5 summarizes expenditure on delivery care. Data were collected from 1204 women as an adjunct to the routine postnatal interview from January to March 2007. The table includes direct and indirect expenditure on normal deliveries, both within and outside Mumbai. Direct expenditure includes doctors' fees, hospital charges and medications. Indirect expenditure mainly describes loss of income, transport and food costs for the woman and her companion during her stay at the hospital. The median cost of a home delivery in Mumbai was around Rs 1000 (US$ 21), the largest tranche of which was the birth attendant's fee. Public sector delivery cost a similar amount, although it rose to around Rs 1500 (US$ 32) if indirect costs were included. The same indirect costs applied to private sector delivery, which was substantially more costly, with a median of Rs 5500 (US$ 118). Costs of home delivery were lower outside Mumbai, by about half. This also applied to private institutional delivery. Reassuringly, public sector delivery costs were similar within and outside the city.

**Table 5:** Expenditure on care for normal delivery in 48 Mumbai slums

Values are Indian Rupee	N	Median	IQR	Range
**Delivery in Mumbai**				
Home delivery*				
TBA fee	73	500	500-600	50-1550
Fluids	3	300	150-500	150-500
Doctor's fee	48	200	100-250	50-700
Medications	22	200	100-300	10-500
Injections	18	175	50-300	50-700
Delivery kit	26	10	6-60	6-500
Other	35	300	150-500	20-876
Total	90	930	550-1260	0-3500
*Institutional delivery*				
Public sector				
Direct	473	1000	500-2000	0-45000
Indirect	473	500	300-860	0-23450
Total	473	1550	950-2641	290-45000
Private sector				
Direct	258	5000	3500-7225	60-35000
Indirect	258	500	200-950	0-10000
Total	258	5510	3950-8100	360-35700
**Outside Mumbai**				
Home delivery*				
TBA fee	60	300	200-500	100-3000
Fluids	7	300	200-1300	150-3000
Doctor fee	25	200	100-400	30-2000
Injections	11	200	80-250	50-400
Medications	16	175	100-400	20-3500
Delivery kit	10	50	6-300	6-800
Other	32	200	115-350	12-5100
Total	84	550	300-1075	0-7290
*Institutional delivery*				
Public sector				
Direct	69	1000	500-1500	0-7000
Indirect	69	400	150-700	0-3000
Total	69	1300	930-2000	50-8000
Private sector				
Direct	62	2780	1500-5000	500-14000
Indirect	62	425	150-650	0-5400
Total	62	3580	1900-5220	550-15400

Data presented for 1204 women for the period January-March 2007.

*Some respondents were unable to break down the costs of delivery into items. Total figures are therefore based on larger samples.

TBA: traditional birth attendant, *dai; *IQR: interquartile range.

## Discussion

In a two-year prospective study of births in 48 urban slum areas of Mumbai, we found wide variation in the proportion of home deliveries. Most of these were assisted by traditional birth attendants, and the direct costs were not substantially less than for public sector institutional births. Home births were more likely for parous poorer women with less education, living in insubstantial homes in slum areas with high rates of migration and hazardous location.

Limits to the study included the sampling frame, cluster size, loss to follow-up, the omission of certain groups such as pavement dwellers, and the methods used to assess poverty. There was a possibility of reporting bias because interviews were done six weeks after delivery. The cost estimates are at best indicative, since women may not themselves have made payments and since recall is likely to have been difficult. A further limitation was that the reasons for home delivery were recorded as open answers to a brief question within a quantitative interview. This makes them potentially superficial and limits our ability to interrogate the drivers of choice. A better understanding would undoubtedly be gained through qualitative methods such as semi-structured interviews, and we are undertaking a range of qualitative work in our current efforts to understand urban health from a women's perspective. We also intend to examine cause-specific mortality in a subsequent analysis, particularly as regards the case-mix of home and institutional deliveries.

Three major reviews of the determinants of maternity service use have been published. The first, by Thaddeus and Maine, [18] introduced the idea of three phases of delay in addressing emergencies that could lead to mortality: delay in deciding to seek care on the part of the individual, the family, or both; delay in reaching an adequate health care facility; and delay in receiving adequate care at the facility. The second review, by Say and Raine, [19] considered inequalities in the use of maternal health care in developing countries. It identified urban-rural differences, with urban women more likely to be attended by a skilled health worker, to give birth in a medical setting, and to have had antenatal care in the first trimester of pregnancy. In a recent review, Gabrysch and Campbell grouped 20 potential determinants of delivery service use under four themes: socio-cultural, perceived benefit of or need for skilled attendance, economic accessibility, and physical accessibility [20]. They revisited the idea of delay in care-seeking to make a distinction between action in emergencies and health care for routine or preventive care.

Inequalities in the distribution of home births highlight the health vulnerabilities of the poorest [13]. The analysis of women's individual and social characteristics showed differentials across 48 clusters, and lower socio-economic status was a predictor of home delivery [10,21-25]. Clusters with higher rates of home delivery tended to have poorer environment and sanitation, contested legal status, and less residential tenure. Many of these areas were located on garbage dumps or reclaimed land. Over half of their housing was of insubstantial construction, with common toilet facilities. Authorized electric supply was available to over half of the population, but the scarcity of water compelled people to purchase it from vendors.

Many of the residents of clusters with particularly high rates of home delivery were migrants from Uttar Pradesh and Bihar states working in the informal sector on daily wages. Literacy rates were low and 68% of women identified themselves as Muslim. More than half had been living in the area for less than five years and did not hold a ration card. Although ration cards are issued to families below the poverty line, we have found that their possession tends to favour more established families, and that the lack of a card is a paradoxical indicator of poverty in slum communities.

In comparison, clusters with fewer home births had higher socio-economic scores and were more permanent settlements with better living conditions. Their populations were relatively stable, with more women identifying themselves as Hindu and higher levels of literacy. The findings are in line with other studies, which suggest that maternal education, socioeconomic status, religion and occupation are associated with place of delivery [7,10,18,20]. The mutually confounding effects of these and other factors are hard to disentangle [20].

In addition to socio-economic status, proximity to municipal health facilities and their perceived quality of services affected the choice of delivery location. Since many women do not have the resources to access private health facilities, they depend largely on the municipal health system for delivery care. Distribution of services may not be in line with need, however. In M East ward, where the proportion of home births is highest, there is one peripheral hospital and two maternity homes; F North ward has one maternity home. These institutions suffer from a shortage of medical professionals and equipment, cannot handle obstetric emergencies and referral to other institutions is common. In comparison, institutional delivery was much commoner in G North ward, despite it having the second lowest tier of socio-economic scores. One of the reasons for this was undoubtedly the presence of a nearby tertiary hospital, itself an incentive to bypass more peripheral institutions [26].

We found that 10% of women opted for home deliveries in Mumbai, but that, of the 20% who delivered elsewhere, 38% delivered at home. These were generally women who had decided to return to their native villages for childbirth, a practice throughout India, and women who had moved to the city more recently and might have stronger links with their natal homes. Cultural norms and inaccessibility of institutions were the most important contributing factors to home delivery outside Mumbai. Perhaps it is surprising that, in spite of being aware of the circumstances in their villages and the risks involved, urban women choose to go back to their families for delivery. Other factors explaining home deliveries included sudden onset or short duration of labour [8,9]. In Mumbai, more than three-quarters of women had planned their delivery and registered at an institution, but sudden onset of labour or unavailability of a companion restricted their choice of place of delivery. The study also provided evidence that the behaviour of staff and poor opinion of the quality of care at institutions were disincentives to institutional delivery. More than half of the women who mentioned this had registered for institutional delivery but ended up giving birth at home. This is probably explained by the fact that registration is an automatic component of antenatal care, rather than a clear statement of intent. Previous experiences also deter women from seeking institutional care, [20] and slum residents often express a preference for home birth in a comfortable and reassuring environment [22].

A recent review of the economic implications of home births in Australia, North America and the United Kingdom notwithstanding, [27] few studies have considered their costs. Expenditure was similar to that described in an analysis of national data from 2004, [25] a little higher than figures from rural Rajasthan, [28] but lower than that found in a study in Delhi [29]. We think that one of the most interesting findings was that economic constraint did not seem to be a major reason for home delivery. Few women reported it and we do not think that there was an incentive to hide it in the interview process. Although user fees are a disincentive to institutional delivery, our finding accords with the literature [18,19,25]. We found that, contrary to the general perception that home births are cheaper, direct expenditure was not substantially less than that of delivering at a public hospital. The requirement for an extra Rs 500 could be limiting for some families and tip the balance away from institutional delivery, but at today's prices we think that this would not be common. Whatever the reality, it is possible that the idea that institutional deliveries could be expensive may have acted as a barrier against accessing health facilities. Other disincentives to institutional delivery include the fact that a woman might have to struggle to find someone to look after the house and other children while she is away, or to accompany her to the hospital. The cost of private sector delivery was considerably higher. The main home delivery expenses were fees paid to *dais*, which included gifts in the form of clothes and groceries. In a number of cases, expenses also included fees for doctors called immediately after delivery to attend to mother and baby.

Most home deliveries were assisted by untrained traditional birth attendants, both in Mumbai and outside. For births outside Mumbai, the presence of extended families meant that relatives were the principal birth attendants more often than they were in the city. Urban nuclear families have less recourse: 22 women who gave birth in Mumbai said that they had delivered entirely alone. For most *dais*, their occupation has been pursued for generations and they have developed their skills by observing their mothers and elders conducting deliveries. However, the notion of a 'traditional' birth attendant in a hyper-urban setting is vague. We have begun a qualitative study to better understand how urban women become *dais*, their perceptions and experiences of their role, the problems they encounter during the birth process and their capability in handling them.

Over a quarter of neonatal deaths are due to infections that can be reduced by hygienic practices at the time of delivery [30]. WHO guidelines recommend five cleans for home births: clean surface, clean hands, clean blade, clean cord care and clean perineum. Our findings - particularly on the use of gloves and cord care - were similar to those of other studies, [8,11] and more encouraging than those of a study in Indore, India [31]. Few women reported severe complications at the time of delivery. One of the possibilities may be that access to facilities deters women at risk from delivering at home, or that immediate medical intervention is provided in case of last minute complications. *Dais *may also not want to take risks and refer mothers at the slightest hint of complications.

## Conclusion

If 54% of Mumbai's 16.4 million residents live in slums, if the range of areas covered by the study is representative, and if our own estimates of crude birth rate are reasonable at 23 per 1000, we would expect over 203 600 annual births to slum dwellers. If an average 16% of these are born at home, our best estimate of home births is over 32 000 across the city's slums. This is a large minority by any standards. What should be done? Our findings imply that home deliveries are not evenly distributed and that they cluster with other markers of vulnerability, including poverty, lack of education, poor housing and water supply, hazardous location and insecurity of tenure. This is hardly surprising, but, along with the finding that cost does not seem to be a primary driver of home birth, it does raise the possibility of targeting. Assuming that the overall trend is toward institutional care, [13] it might be productive to focus inputs on more vulnerable areas. We support the removal of user fees from public sector health services, but it is not clear to us that an incentive for institutional delivery is necessary across the board. An alternative strategy might be to concentrate on intensive outreach in vulnerable areas by community-based health workers who could help women to plan their deliveries and make sure that they get help in time; and on efforts to improve the client experience at public sector institutions.

## Competing interests

The authors declare that they have no competing interests. SD had full access to all the data in the study and had final responsibility for the decision to submit for publication.

## Authors' contributions

SD conceived the study, analysed the data and prepared the first draft of the manuscript. UB helped to design the study, supervised data collection and commented on drafts of the manuscript. NSM coordinated the project, helped to design the study and commented on drafts of the manuscript. LC supervised data entry and cleaning, generated analytical outputs and commented on drafts of the manuscript. WJ is the director of SNEHA, had overall responsibility for Mumbai research activities, and commented on drafts of the manuscript. DO helped to design the study, advised on analysis and co-wrote later drafts of the manuscript. All authors contributed to critique and modification of the manuscript and read and approved the final version.

## Role of the funding source

The sponsors had no role in the study design, data collection, analysis, interpretation or writing of the article.

## Pre-publication history

The pre-publication history for this paper can be accessed here:

http://www.biomedcentral.com/1471-2393/10/38/prepub
